# MITRA-FR vs. COAPT: lessons from two trials with diametrically opposed results

**DOI:** 10.1093/ehjci/jez073

**Published:** 2019-04-23

**Authors:** Philippe Pibarot, Victoria Delgado, Jeroen J Bax

**Affiliations:** 1 Institut Universitaire de Cardiologie et de Pneumologie de Québec/Québec Heart and Lung Institute, Department of Medicine, Université Laval, 2725 Chemin Sainte-Foy, Pavillon A Québec city, Québec, Canada; 2 Department of Cardiology, Leiden University Medical Center, Albinusdreef 2, RC Leiden, The Netherlands

**Keywords:** transcatheter, mitral regurgitation, heart failure, echocardiography

## Abstract

Percutaneous mitral valve repair using the MitraClip device has been proposed to correct secondary mitral regurgitation (MR). Recently, the results of two randomized controlled trials, that is MITRA-FR (Percutaneous Repair with the MitraClip Device for Severe Functional/Secondary Mitral Regurgitation) and COAPT (Cardiovascular Outcomes Assessment of the MitraClip Percutaneous Therapy for Heart Failure Patients with Functional Mitral Regurgitation), assessing the efficacy and safety of MitraClip in patients with systolic heart failure and severe secondary MR were published. A priori, these two trials targeted the same patient populations with the same disease using the same device but the results of these trials were diametrically opposed, MITRA-FR being neutral and COAPT being highly positive with respect to efficacy of the MitraClip procedure. The objectives of this viewpoint are: (i) to highlight not only the similarities but also the differences between MITRA-FR and COAPT, which may explain the strikingly different results and conclusions between these two trials and (ii) to derive from these results, implications with regards to the application of the MitraClip procedure in clinical practice.

## Introduction

The overall prevalence of mitral regurgitation (MR) in the general population is ∼2% and its aetiology may be primary (or organic) or secondary (or functional). Secondary MR is a consequence of annular dilatation and geometrical distortion of the sub-valvular apparatus secondary to left ventricular (LV) remodelling associated with cardiomyopathy or coronary artery disease. Severe secondary MR is associated with a poor prognosis in patients with chronic heart failure and reduced left ventricular ejection fraction (LVEF). Percutaneous mitral valve repair using the MitraClip device has been proposed to correct secondary MR. Recently, the results of two randomized controlled trials, that is MITRA-FR (Percutaneous Repair with the MitraClip Device for Severe Functional/Secondary Mitral Regurgitation) and COAPT (Cardiovascular Outcomes Assessment of the MitraClip Percutaneous Therapy for Heart Failure Patients with Functional Mitral Regurgitation), assessing the efficacy and safety of MitraClip in patients with systolic heart failure and severe secondary MR were published in the *New England Journal of Medicine*.^1^^,^^2^ A priori, these two trials targeted the same patient populations with the same disease using the same device but the results of these trials were diametrically opposed, MITRA-FR being neutral and COAPT being highly positive with respect to efficacy of the MitraClip procedure.

The objectives of this viewpoint are: (i) to highlight not only the similarities but also the differences between MITRA-FR and COAPT, which may explain the strikingly different results and conclusions between these two trials and (ii) to derive from these results, implications with regards to the application of the MitraClip procedure in clinical practice.

## Summary of the design and results of MITRA-FR and COAPT

The *MITRA-FR* study randomized 304 patients with symptomatic systolic heart failure and severe secondary MR defined as an effective regurgitant orifice area (EROA) >20 mm^2^ and/or a regurgitant volume >30 mL, and LVEF between 15% and 40%, in a 1:1 ratio, to percutaneous mitral valve repair with MitraClip in addition to optimized medical therapy (intervention group) or to optimized medical therapy alone (control group) (*Tables 1*and***2*).^1^ The primary efficacy endpoint was a composite of death from any cause or hospitalization for heart failure at 1 year. There was no difference between the intervention vs. control groups for the rate of the primary composite endpoint (54.6% vs. 51.3%, respectively; *P* = 0.53), the rate of mortality (24.3% vs. 22.4%) or the rate of unplanned heart failure hospitalization (48.7% vs. 47.4%) (*Table 3*). The authors concluded that MitraClip is safe and effective in reducing secondary MR but does not improve prognosis (as compared with optimized medical therapy) in patients with secondary MR and systolic heart failure.

**Table 1:** Similarities and differences among MITRA-FR, COAPT, and RESHAPE-HF2 with respect to study design and endpoints

	MITRA-FR	COAPT	RESHAPE HF2
Study design	Prospective, randomized	Prospective, randomized	Prospective, randomized
Randomization 1:1 in:			
Intervention arm	MitraClip + GDMT	MitraClip + GDMT	MitraClip + GDMT
Control arm	GDMT	GDMT	GDMT
Patientsrecruitment			
Total no. of patients	304	614	420
No. of patients in intervention/control groups	152/152	302/312	
Enrolment period, year	3.2	4.8	
No. of sites	22	85	
No. of patients/site	8.2	7.8	
No. of patients/site/year	2.6	1.6	
Inclusion/exclusion criteria	By European Guidelines^6^	By American Guidelines^8^^,^^12^	By EACVI recommendations^12^	
≥ Moderate-to-severe (3+) MR	EROA >20 mm^2^ and/or RV >30 mL	EROA ≥30 mm^2^ and/or RV >45 mL	EROA >30 mm^2^ and/or RV >45 mL	
LV end-systolic diameter, mm		≤70 mm		
LV ejection fraction, %	≥15 and ≤40	≥20 and ≤50	≥15 and ≤35 if NYHA II≥15 and ≤45 if NYHA III or IV	
GDMT at baseline	GDMT variable adjustment in each group per ‘real-world’ practice	Stable maximal doses of GDMT and cardiac resynchronization therapy (if appropriate)	Stable optimal GDMT and revascularization or cardiac resynchronization therapy (if appropriate)
Symptomatic status	NYHA class: II, III, IV	NYHA class: II, III, IVa (ambulatory)	NYHA class: II, III, IV
Surgical risk	Not candidate for mitral-valve surgery	Not candidate for mitral-valve surgery	Mitral-valve surgery is not the preferred option
Primary endpoint	Death or HF hospitalization at 1 year	HF hospitalization at 1 year	Composite rate of recurrent HF hospitalizations and cardiovascular death at 2 years

EACVI, European Association of Cardiovascular Imaging; EROA, effective regurgitant orifice area; GDMT, guideline-directed medical therapy; HF, heart failure; MR, mitral regurgitation; NYHA, New York Heart Association; RV, regurgitant volume.

**Table 2:** Similarities and differences between MITRA-FR and COAPT with respect to baseline characteristics of the study populations

	MITRA-FR	COAPT
Baseline clinical characteristics		
Age, year	70 ± 10	72 ± 11
NYHA class, %		
I	0	0.2
II	32.9	39.0
III	58.5	52.5
IV	8.6	8.3
Surgical risk		
STS score ≥8%		42.7%
EuroSCORE II, median and IQR	6.2 (3.5–11.0)	
Baseline echocardiographic characteristics		
MR severity, %		
Moderate (EROA 20-29 mm^2^)	52	14
Moderate-to-severe (EROA 30-39 mm^2^)	32	46
Severe (EROA ≥ 40 mm^2^)	16	41
EROA, mm^2^	31 ± 10	41 ± 15
LV end-diastolic volume index, mL/m^2^	135 ± 35	101 ± 34
LV ejection fraction, %	33 ± 7	31 ± 9

IQR, inter-quartile range; STS, Society of Thoracic Surgery. Other abbreviations as in *Table 1*.

**Table 3:** Similarities and differences between MITRA-FR and COAPT with respect to study outcomes

	MITRA-FR	COAPT
Procedural characteristics and outcomes^a^		
Procedural success, %^a^	96	98
Procedural complications, %^a^	14.6	8.5
Number of clips, %^b^		
1 Clip	46	36
2 Clips	45	55
3 Clips	9	8
4 Clips	0	0.3
Post-procedural MR ≥ moderate-to-severe (3+), %^a^		
End of procedure	9	5
1 year post-procedure	17	5
2 years post-procedure		0.9
1-Year outcomes		
1-Year mortality, %		
Intervention	24.2	19.1 (*P* < 0.001)
Control	22.4	23.2
1-Year heart failure hospitalization, %		Primary outcome
Intervention	48.7	35.8 (*P* < 0.001)^c^
Control	47.4	67.9^c^
1-Year mortality or heart failure hospitalization	Primary outcome	
Intervention	54.6 (*P* = 0.53)	33.9 (*P* < 0.001)
Control	51.3	46.5

Abbreviations as in *Table 1*.

a Data are from the intervention group only.

b Data are from the intervention group with procedural success.

c Annualized rate (in % per year) within 2-year follow-up.

The *COAPT* trial randomly assigned 614 patients with symptomatic heart failure and moderate-to-severe or severe secondary MR defined as an EROA >30 mm^2^ and/or regurgitant volume >45 mL, and LVEF ≥20%, in a 1:1 ratio, to percutaneous mitral valve repair with MitraClip plus optimized medical therapy (intervention group) or to optimized medical therapy alone (control group) (*Tables 1 and **2*).^3^ The primary efficacy endpoint was all hospitalizations within 2-year follow-up. The annualized rate of all hospitalizations for heart failure within 2 years was 35.8% per patient-year in the intervention group as compared with 67.9% per patient-year in the control group (*P* < 0.001) (*Table 3*). Death from any cause within 2 years occurred in 29.1% of the patients in the intervention group vs. 46.1% in the control group (*P* < 0.001). The authors concluded that among patients with heart failure and ≥ moderate-to-severe secondary MR who remained symptomatic despite the use of optimal guideline-directed medical therapy (GDMT), the MitraClip procedure reduces the rates of hospitalization for heart failure and all-cause mortality within 2 years of follow-up than medical therapy alone. The number needed to treat to prevent one hospitalization for heart failure within 24 months was 3.1.

### Similarities and differences between MITRA-FR and COAPT

*Tables* 1–3 present the main similarities and differences in study design and results between the MITRA-FR and COAPT trials. The sample size was about 2-fold larger in the COAPT study vs. the MITRA-FR trial and the primary efficacy endpoint was the rate of the composite of death from any cause and unplanned hospitalization for heart failure at 1 year in the MITRA-FR trial vs. the rate of all hospitalizations for heart failure within 2-year follow-up in the COAPT study.

## Extent of LV damage and MR severity

Compared with patients in the COAPT trial, those enrolled in the MITRA-FR trial had substantially more LV damage. The patients had larger LV end-diastolic volumes (MITRA-FR: 135 ± 35 mL/m^2^ vs. COAPT: 101 ± 34 mL/m^2^) suggesting a more advanced stage of the LV disease (*Table 2*). This difference is likely related to the fact that COAPT excluded patients with very severe LV dilation/dysfunction (LV end-systolic diameter <70 mm), whereas MITRA-FR had no LV dimension limit. Also in COAPT, the inclusion criteria for LVEF were 20–50% vs. 15–40% in MITRA-FR. Several studies have reported that in heart failure patients with ischaemic MR, severe LV dilation (LV end-diastolic diameter >65 mm) and LV dysfunction (LVEF < 20%, LV end-systolic diameter >55 mm) are associated with high rates of persistent/recurrent MR, less reverse LV remodelling, and worse outcomes after surgical correction of ischaemic MR.^4^^,^^5^

MITRA-FR patients also had less severe MR (EROA: 31 ± 10 mm^2^) as compared with COAPT (41 ± 15 mm^2^) (*Table 2*). Although the inclusion criteria were at least moderate-to-severe (3+) secondary MR in both trials, MITRA-FR actually used the 2012 European guidelines criteria,^6^ that is EROA ≥20 mm^2^ and/or regurgitant volume ≥30 mL, whereas COAPT used the 2006/2008 American guidelines criteria,^7^^,^^8^ that is EROA ≥30 mm^2^ and/or regurgitant volume ≥45 mL. The criteria used in MITRA-FR, correspond to ≥ moderate (or 2+) MR according to American guidelines criteria.^7^^,^^8^ The European guidelines^6^ as well as the 2014 American guidelines^9^ recommended to use 2-fold lower cut-off values of EROA (20 vs. 40 mm^2^) and regurgitant volume (30 vs. 60 mL) to define severe MR in secondary vs. primary MR. This was based on the rationale that the risk of mortality rises significantly at a lower level of MR severity (EROA ≥20 vs. 40 mm^2^) in secondary vs. primary MR.^10^^,^^11^ However, ischaemic MR is a complex and multifaceted disease and it is unclear whether a volumetrically moderate MR is truly an actor or simply a marker of the LV adverse remodelling/dysfunction and of the heart failure symptoms; in other words whether it is primarily a valvular disease or a myocardial (LV) disease. If one applies the same criteria of EROA to define the severity of MR, there appears to be major difference in the distribution of baseline MR severity between MITRA-FR and COAPT: only 16% of MITRA-FR patients had severe MR as defined by EROA ≥40 mm^2^ vs. 41% of COAPT patients (*Table 2*).

It could be that these differences in the inclusion/exclusion criteria for MR severity, LV dimensions and dysfunction are the main reasons for the discrepancies in the outcomes observed between MITRA-FR and COAPT (*Table 1*). In MITRA-FR, the underlying cardiomyopathy (myocardial or LV disease) was likely the predominant cause of the heart failure and thus the main determinant of the poor clinical outcome. And in this context, the MR was probably more a bystander than an actor of the heart failure. On the other hand, in COAPT, heart failure was, in large part, related to the valvular disease (the MR was more severe), while LV disease (smaller size and higher LVEF) was less advanced. Hence in COAPT, MR was an important contributor to the heart failure and the clinical outcomes, whereas in MITRA-FR the LV disease (dysfunction) was the main determinant of clinical outcomes. Possibly, because of these differences in baseline characteristics, the COAPT patients were more likely to benefit from the MitraClip procedure compared with the MITRA-FR patients.

## Optimization of medical therapy at baseline and during follow-up

To confirm patient eligibility, both trials required that patients remained symptomatic (NYHA class 2 or more) despite the use of GDMT for chronic systolic heart failure (*Table 1*). However, COAPT imposed a more demanding criteria for inclusion of patients: that is use of maximal tolerated doses of GDMT, and treatment with cardiac resynchronization therapy, defibrillators, and revascularization, if appropriate. Hence in COAPT, medical treatment was optimized prior to randomization and only a few major adjustments in treatment occurred during follow-up. On the other hand in MITRA-FR, medical therapy was not optimized in all patients at baseline and multiple adjustments in medical treatment were allowed in each arm during follow-up per ‘real-world’ practice. This issue may also have decreased the ability to reveal a beneficial effect of the intervention in MITRA-FR.

## Efficacy in the correction of MR

A more aggressive strategy for correction of MR was applied in COAPT, as suggested by the larger number of clips implanted per patient in COAPT vs. in MITRA-FR (*Table 3*). Furthermore, the rate of sustained reduction of MR was higher in COAPT than in MITRA-FR. At 1 year, 17% of the MITRA-FR patients randomized to MitraClip had ≥ moderate-to-severe (3+) residual MR compared with only 5% in COAPT. The lower sustained efficacy of the MitraClip procedure may also have contributed to the lack of benefit of the intervention in MITRA-FR.

## Conclusions and implications for the management of patients with secondary MR

**Figure 1:**
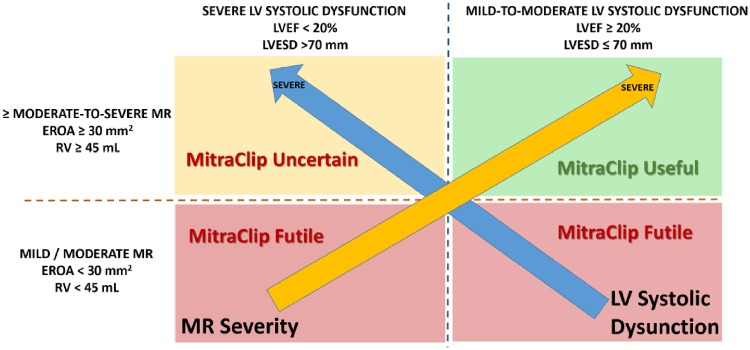
Utility vs. futility of MitraClip procedure according to severity of MR and LV systolic dysfunction. EROA, effective regurgitant orifice area; LVEF, left ventricular ejection fraction; LVESD, left ventricular end-systolic diameter; MR, mitral regurgitation; RV, regurgitant volume.

MITRA-FR and COAPT targeted the same disease entity with the same device, the MitraClip. However, COAPT enrolled a subset of patients who had more severe MR and less advanced LV disease (dilation/dysfunction) compared with MITRA-FR patients. These differences may explain the different outcomes observed in COAPT vs. MITRA-FR. Indeed, patients with too severe LV dilation/dysfunction (i.e. too extensive LV myocardial damage) may not benefit from the MitraClip procedure (*Figure 1*).

In light of the results of the MITRA-FR and COAPT trials, it thus appears reasonable to conclude that the MitraClip procedure reduces heart failure hospitalization and mortality in patients meeting the following criteria (*Figure 1*):

1. ≥ moderate-to-severe (3+) secondary MR defined as EROA ≥30 mm^2^ and/or regurgitant volume >45 mL;
2. LVEF between 20% and 50% and LV end-systolic diameter <70 mm;
3. Persistent heart failure symptoms (NYHA ≥ II) despite optimal (maximally tolerated) GDMT with cardiac resynchronization and coronary revascularization if appropriate.

Furthermore, the goal of the procedure should be to obtain an acute reduction of the MR severity to ≤ mild (1+) and the implantation of additional clips should be considered to achieve this goal.

Further insight will come from the results of the ReshapeHF2 trial [A Clinical Evaluation of the Safety and Effectiveness of the MitraClip System in the Treatment of Clinically Significant Functional Mitral Regurgitation (Reshape-HF2) (https://clinicaltrials.gov/ct2/show/NCT 02444338], which has the same inclusion criteria as those of the COAPT trial in terms of MR severity, with intermediary criteria COAPT and MITRA-FR in terms of LV dysfunction severity (*Table 1*).

## Funding

P.P. holds the Canada Research Chair in Valvular Heart Disease, Canadian Institutes of Health Research (CIHR), Ottawa Canada and received Foundation Scheme research grant #FDN-143225) from CIHR. The department of Cardiology of the Leiden University Medical Center receives unrestricted research grants from Biotronik, Boston Scientific, Medtronic, Edwards Lifesciences and GE Healthcare.

**Conflict of interest:** P.P. received funding from Edwards Lifesciences and Cardiac Phoenix for echocardiography corelab analyses with no personal compensation. J.J.B. received speaker fees from Abbot Vascular and Boehringer Ingelheim. V.D. received speaker fees from Abbott Vascular.
